# Direct-methods structure determination of a trypanosome RNA-editing substrate fragment with translational pseudosymmetry

**DOI:** 10.1107/S2059798316001224

**Published:** 2016-03-24

**Authors:** Blaine H. M. Mooers

**Affiliations:** a Department of Biochemistry and Molecular Biology, and Stephenson Cancer Center, University of Oklahoma Health Sciences Center, 975 NE 10th Street, BRC 466, Oklahoma City, OK 73104, USA

**Keywords:** RNA structure determination, Patterson analysis, helical symmetry, *ab initio* phasing, noncrystallographic symmetry

## Abstract

Using direct methods starting from random phases, the crystal structure of a 32-base-pair RNA (675 non-H RNA atoms in the asymmetric unit) was determined using only the native diffraction data (resolution limit 1.05 Å) and the computer program *SIR*2014. The almost three helical turns of the RNA in the asymmetric unit introduced partial or imperfect translational pseudosymmetry (TPS) that modulated the intensities when averaged by the *l* Miller indices but still escaped automated detection. Almost six times as many random phase sets had to be tested on average to reach a correct structure compared with a similar-sized RNA hairpin (27 nucleotides, 580 non-H RNA atoms) without TPS. More sensitive methods are needed for the automated detection of partial TPS.

## Abstract 2

The crystal structure of a 32-base-pair RNA double helix (675 non-H atoms) from a trypanosome RNA-editing substrate was determined with 1.05 Å resolution X-ray diffraction data starting from random phases using the direct-methods computer program *SIR*2014. Success was achieved in the presence of two levels of translational pseudosymmetry caused by three helical repeats.

## 1. Introduction   

Automated structure determinations by direct methods of equal-atom proteins (*i.e.* atoms lighter than calcium) with 1000 non-H protein atoms have been achieved when starting from random phase angles (*i.e.* *ab initio* direct methods), when using dual-space methods and when the diffraction data extend to atomic resolution (Sheldrick, 1990 ▸; Morris & Bricogne, 2003 ▸; Langs & Hauptman, 2011 ▸; Giacovazzo, 1998 ▸, 2014 ▸). These constraints are relaxed when calcium or heavier atoms are present, when Patterson superposition methods are used or when Patterson methods and heavy atoms are used together (Burla *et al.*, 2006 ▸; Caliandro *et al.*, 2007 ▸, 2008 ▸; Mooers & Matthews, 2004 ▸, 2006 ▸). We found only one report of *ab initio* direct methods being successfully applied to an unknown RNA molecule (Safaee *et al.*, 2013 ▸). Success in direct-methods structure determination could be expected to be easier with nucleic acids than with proteins because the P atoms in the backbone of RNA are electron-dense, even though they are sometimes in two alternate conformations (Luo *et al.*, 2014 ▸), and because the P atoms occur at a higher frequency (∼1 in 20) in nucleic acids than S atoms occur in proteins (1 in 100–300; Ramagopal *et al.*, 2003 ▸). On the other hand, translational pseudosymmetry (TPS) caused by helices longer than one turn may inhibit structure determination by direct methods because the internal symmetry violates the assumption that the atoms in the asymmetric unit are randomly distributed. This idea is supported by many reports of TPS hindering the direct-methods structure determination of small-molecule crystal structures and the molecular-replacement structure determination of proteins (Dauter *et al.*, 2005 ▸). The role of TPS in phasing has been explored many times in chemical crystallography and is a current interest in biological crystallography (Hauptman & Karle, 1959 ▸; Böhme, 1982 ▸; Gramlich, 1984 ▸; Cascarano *et al.*, 1985 ▸, 1987 ▸; Fan, Qian *et al.*, 1988 ▸; Fan, Yao *et al.*, 1988 ▸; Zwart *et al.*, 2008 ▸; Read *et al.*, 2013 ▸). The rational dependence of the atoms related by TPS leads to sets of strong reflections and weak reflections. Most of the phase relationships depend on strong reflections if the presence of TPS is ignored. The weak reflections can be used to form separate phase relationships (Cascarano *et al.*, 1988*a* ▸,*b* ▸; Fan, Qian *et al.*, 1988 ▸; Fan, Yao *et al.*, 1988 ▸). Rotational pseudosymmetry in crystal structures of short dsRNAs has been reported (Kondo *et al.*, 2008 ▸) and the prospects for direct methods with oligonucleotides shorter than one helical turn have been explored (Hubbard *et al.*, 1994 ▸), but we know of no published applications of direct methods to nucleic acids with TPS present. The most common TPS in protein crystals involves two molecules in the asymmetric unit. Sometimes TPS is found within a single protein; three of 1007 protein superfamilies have internal TPS (Myers-Turnbull *et al.*, 2014 ▸). In contrast, RNA double helices longer than one helical turn could have imperfect TPS caused by the helical repeats. This TPS could restrict success in direct methods to RNAs of one helical turn in length or shorter. Previous nucleic acid structures determined *ab initio* by direct methods have been one helical turn long or shorter (Egli *et al.*, 1998 ▸; Han, 2001 ▸; Safaee *et al.*, 2013 ▸; Luo *et al.*, 2014 ▸).

We tested the idea that *SIR*2014 could still determine the structure of a dsRNA with imperfect TPS by *ab initio* direct methods without detecting the TPS. We compared the direct-methods structure determination of a double-stranded RNA (dsRNA; 32 base pairs, one strand in the asymmetric unit, 675 non-H atoms, two levels of imperfect TPS) with that of a single-stranded RNA (ssRNA) hairpin (27 nucleotides, one strand in the asymmetric unit, 580 non-H RNA atoms, no TPS). The dsRNA is a pathological case for *ab initio* structure determination in the presence of TPS and the hairpin is a case for *ab initio* structure determination by direct methods in the absence of TPS. The *ab initio* structure-determination experiments were performed with the direct-methods program *SIR*2014, which uses dual-space methods to attempt structure determination from random phases. Owing to the stochastic nature of the phasing process (*i.e.* starting from different sets of random phases in each trial), the number of failed trials before success in one phasing experiment says little about the next phasing experiment that tests a different series of random phase sets. Therefore, a large number of phasing experiments were conducted to obtain the empirical probability mass function (pmf) of success with each data set. The pmf for the dsRNA was broader than that for the hairpin and the mean number of trials was almost six times larger. To investigate this difference, we compared the intensity distributions, Patterson maps, the translation vectors used to shift misplaced trial structures and the effect of removing the strongest reflections on success in structure determination. The presence of TPS enhanced the strong intensities and made the loss of the strongest intensities a larger problem. Our results should appeal to workers interested in phasing methods, RNA crystallography or both.

## 2. Materials and methods   

### 2.1. Construct design, crystallization and data collection   

The design, crystallization, X-ray diffraction data collection, structure determination and structure description of the hairpin RNA (PDB entry 3dw4) have previously been published (Olieric *et al.*, 2009 ▸). The related structure factors were retrieved from the Protein Data Bank. This hairpin is from the sarcin/ricin domain of the *Escherichia coli* 23S RNA (Olieric *et al.*, 2009 ▸). The same experimental aspects of the dsRNA will be described in detail elsewhere, so they are only summarized here. Two 16-base pair U-helix domains from a RNA-editing substrate in trypanosomes were fused head-to-head to promote duplex formation by the 3′ tail of 16 Us that would otherwise form a random coil with unstacked bases in solution at room temperature (Mooers & Singh, 2011 ▸). The fusion RNA was made by phosphoramidite chemistry and was gel-purified to single-nucleotide resolution (Dharmacon, GE Healthcare). Crystals were grown at room temperature from 50 m*M* sodium cacodylate pH 6.5, 20–50 m*M* MgCl_2_, 1–2 *M* lithium sulfate. The crystals were cryoprotected by passage through 1.9, 2.4 and 2.9 *M* sodium malonate pH 6.0. (There was no evidence of arsenic in the X-ray fluorescence scans of similarly treated crystals because the sodium malonate had displaced the cacodylate molecules in the crystal.) X-ray diffraction data were collected on beamline 7-1 at SSRL with 0.979 Å wavelength radiation and an ADSC Quantum 315r detector. The diffraction data were collected at four distances between the detector and the crystal to properly measure the very strong reflections at medium resolution associated with the base stacking in the RNA. The long *c* edge of the unit cell was manually aligned within 40° of the rotation axis of the crystal to avoid spot overlap at high resolution. About 40 crystals with a longest dimension of 0.2–0.4 mm were screened for diffraction quality. Most crystals diffracted X-rays to between 1.4 and 1.2 Å resolution, but one crystal diffracted X-rays to 1.05 Å resolution and was selected for data collection. The diffraction data were processed with *iMOSFLM* (Battye *et al.*, 2011 ▸) and *SCALA* (Evans, 2006 ▸). Data-collection statistics are reported in Table 1 ▸.

### 2.2. Direct-methods structure-determination experiments   

The merged native data for the dsRNA were used with the computer program *SIR*2014 (Burla *et al.*, 2015 ▸) running on individual central processing units (CPUs) on a Xeon64 octa-core Linux cluster in the Oklahoma Center for Supercomputing Education and Research (OSCER) at the University of Oklahoma. Each CPU executed an independent experiment that tested up to 600 different sets of random phases. Each structure-determination trial started with a different set of phases. The phases were pseudorandom numbers that could be recreated by specifying the index of the phase set. The *SIR*2014 code was not parallelized for the execution of one phasing trial on multiple CPUs or on graphical processing units. The modern direct-methods (MDM) phasing protocol in *SIR*2014 was used with its default parameters. The *RELAX* procedure was available to all phasing trials; this protocol shifted to the correct origin promising phase sets that were developing near the wrong origin (Burla *et al.*, 2000 ▸; Caliandro *et al.*, 2007 ▸). To find the shift vector, the diffraction data were expanded to *P*1. After the shift vector was located in *P*1, the program returned to the original space group. The same structure-determination procedure was used with the hairpin RNA.

### 2.3. Automated model building and refinement   

The first correct *ab initio* phases for the dsRNA and the hairpin RNA were used in automated model building with *Nautilus* (Cowtan, 2014 ▸). The models from *Nautilus* were corrected manually using *Coot* (Emsley *et al.*, 2010 ▸). The *RCrane* plugin for *Coot* was used with the hairpin RNA, which required extensive correction owing to the presence of several non-Watson–Crick base pairs (Keating & Pyle, 2012 ▸). The refinement of each model was started at the resolution limit using stereochemistry restraints derived from atomic resolution crystal structures of nucleotides, *PHENIX* and all of the diffraction data (Parkinson *et al.*, 1996 ▸; Adams *et al.*, 2010 ▸). The *REEL* program within *PHENIX* was used to generate stereochemical restraints for the O2′-methyluridine found at position 2650 in the hairpin RNA (Moriarty *et al.*, 2009 ▸). The refinements were initiated with isotropic atomic displacement parameters (ADPs) and no H atoms. Large drops in *R*_free_ on the change to anisotropic ADPs justified replacing the isotropic ADPs with anisotropic ADPs. Likewise, smaller but still significant drops in *R*_free_ warranted the addition of H atoms. The final refinement statistics are reported in Table 2 ▸. The final structures (dsRNA, PDB entry 5da6; redetermined hairpin RNA, PDB entry 5d99) have been deposited in the Protein Data Bank and the Nucleic Acid Database (Berman *et al.*, 2000 ▸).

## 3. Results   

**Figure 1:**
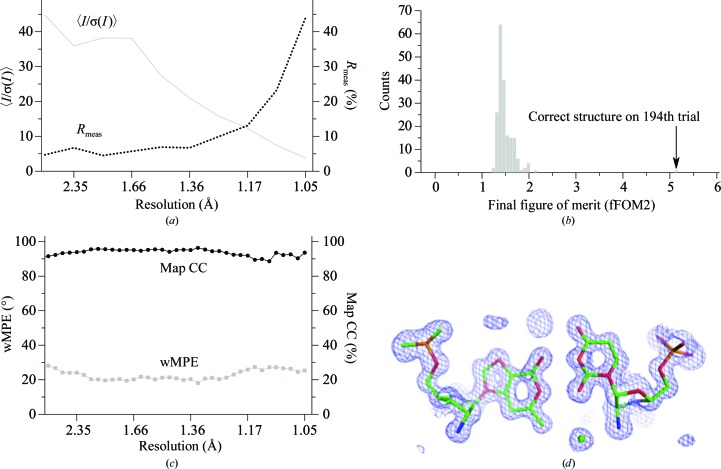
Initial *ab initio* structure determination of the 32-nucleotide dsRNA by direct methods using *SIR*2014 (PDB entry 5da6). (*a*) Data quality as indicated by *R*_meas_ and the 〈*I*/σ(*I*)〉 signal-to-noise ratio. (*b*) The distribution of the final figure of merit (fFOM2) for the 194 phasing trials in the first experiment. (*c*) The weighted mean phase error (wMPE) *versus* resolution and the map correlation coefficient (mapCC) *versus* resolution for the winning phase set in (*b*) (Lunin & Woolfson, 1993 ▸). The final refined model served as the source of the ‘true’ phases. (*d*) *F*_o_exp(*SIR*2014 phases) electron-density map for dsRNA with the model from automated peak picking without knowledge of the RNA stereochemistry. The atom types are colored as follows: carbon, green; nitrogen, blue; oxygen, red; phosphorus, orange. The map was rendered with *PyMOL*.

**Figure 2:**
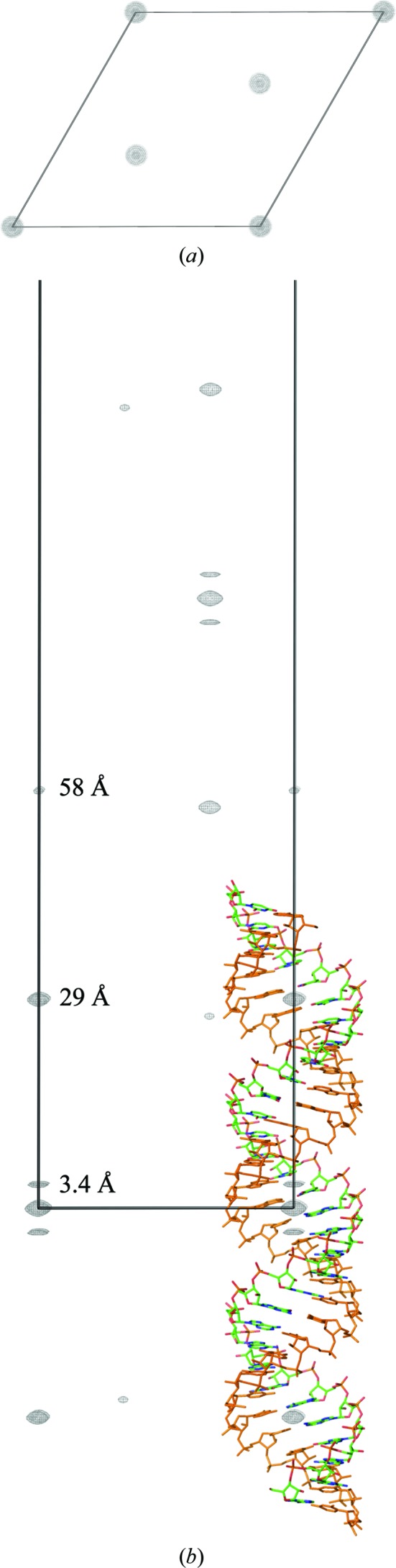
Native Patterson map of the dsRNA obtained with 1.05 Å resolution diffraction data (PDB entry 5da6). (*a*) Map at the *w* = 0 level contoured at the 12σ level. (*b*) Map at the *u* = 0 level contoured at the 12σ level. A stick model of the biological unit without the solvent is overlaid on the unit-cell origin. The single-colored strand was generated by crystallographic symmetry. The off-origin peak at 29 Å corresponds to the length of one helical turn and the peak at 58 Å corresponds to the length of two helical turns.

We compared the structure determinations of dsRNA with three helical turns and of a hairpin with one helical turn and thus no TPS. The diffraction data for the dsRNA were 99% complete (Table 1 ▸) and had a resolution limit of 1.05 Å (Fig. 1 ▸ *a*). The native Patterson map showed evidence of TPS (Fig. 2 ▸). The hairpin RNA was the closest in size to the 32 nt RNA of the available RNA structures with diffraction data at similar resolution. Its diffraction data were nearly complete, and the structure lacked calcium or heavier atoms. Next, we describe the initial structure determination of the dsRNA. The same structure-determination procedure was used with the data from the 27 nt hairpin RNA. We compared the distribution of the number of failed trials before a correct structure for the dsRNA and the hairpin, and found a large difference. We also found differences in the distributions of the intensities and of the vectors used to shift misplaced trial structures. In addition, we found a difference in the sensitivity to the removal of the strongest reflections. The details of the structure of the dsRNA are irrelevant to the central question of this paper and will be described elsewhere. Because each case has a sample size of one, the results reported below cannot be used to make inferences about the ease of structure determination by direct methods with diffraction data from other RNAs.

### 3.1. TPS in the asymmetric unit   

**Figure 3:**
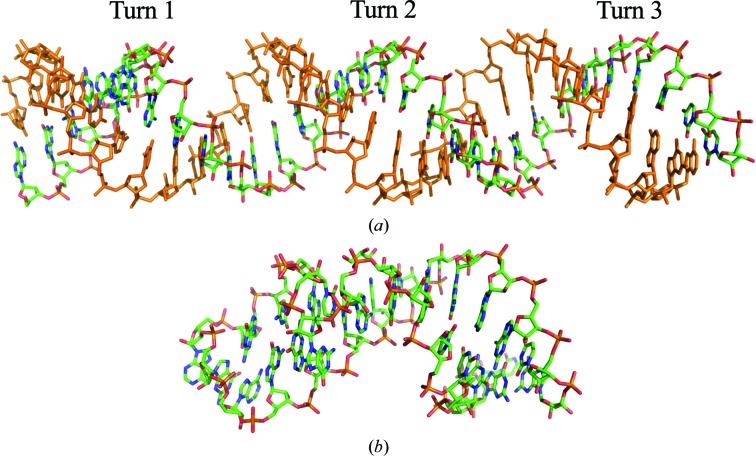
Comparison of the final structures of (*a*) the dsRNA (PDB entry 5da6) and (*b*) the hairpin RNA (PDB entry 3wd4). The single-colored strand in (*a*) was generated by crystallographic symmetry.

The 32 nt dsRNA was a head-to-head fusion of two U-helices from a RNA-editing substrate from trypanosomes (Mooers & Singh, 2011 ▸). The 3′ half of the fusion RNA consisted of 16 consecutive Us that represented the U-tail of the guide RNA. This tail formed ten A–U Watson–Crick base pairs and six G–U wobble base pairs with the 5′ half of the fusion RNA. The 5′ half represented the purine-rich pre-edited mRNA. The RNA was one base pair short of the 33 base pairs required for three helical turns in A-form RNA (11 base pairs per turn). Three double helices stacked end-on-end along the *c* edge of the *R*32:*H* unit cell. One strand was in the asymmetric unit (colored by atom type in Fig. 2 ▸ *b*). The base pairs were inclined by about 16° with respect to the *c* edge of the unit cell. Strong peaks appeared 3.4 Å from the origin in the native Patterson map; these peaks corresponded to parallel, interatomic vectors between adjacent base pairs (Fig. 2 ▸). The interatomic vectors between a base pair and its next-nearest neighbor were much weaker. A peak with a height 57.2% of that of the origin peak was located at a distance of 29 Å from the origin along the *w* edge of the Patterson map. This distance corresponded to the length of one helical turn (Fig. 2 ▸ *b*). Translation vectors between atoms in turns 1 and 2 (r.m.s.d. of 1.3 Å for backbone atoms only) and between atoms in turns 2 and 3 (r.m.s.d. of 1.3 Å for backbone atoms) (Fig. 3 ▸ *a*) gave this peak a double weight. A smaller peak along *w* at 58 Å (Fig. 2 ▸ *b*) from the origin was caused by the vectors between turns 1 and 3 (r.m.s.d. of 1.7 Å for backbone atoms). These longer vectors were half of the number of the vectors that made the higher peak and lead to a second off-origin peak less than half the height of the first peak. The dsRNA crystal structure with the second turn deleted gave a calculated Patterson map that lacked the first peak. Likewise, the crystal structure with the third turn deleted gave a calculated Patterson map that lacked the second peak. These calculated maps validate our interpretation of the native Patterson map. The TPS was too imperfect to be detected automatically by the algorithm used by *SIR*2014.

**Figure 4:**
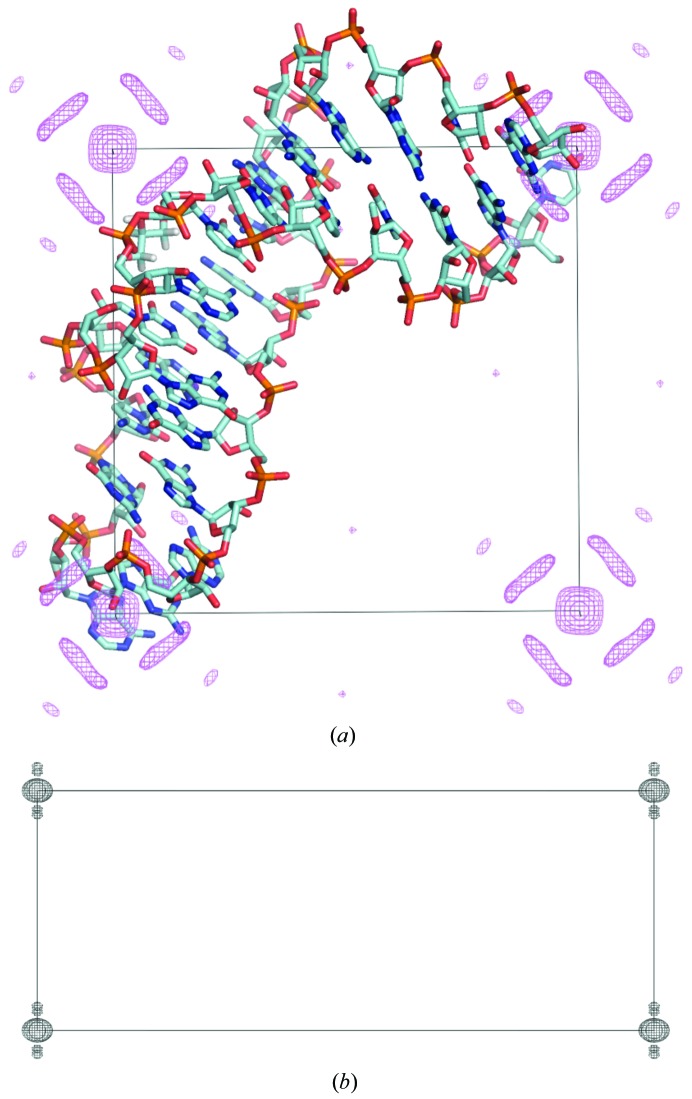
Native Patterson map generated from the 0.97 Å resolution diffraction data of the hairpin RNA (PDB entry 3wd4). (*a*) Map generated with all of the data contoured at the *w* = 0 level and contoured at the 3σ level. (*b*) The same map contoured at the 12σ level. A stick model of the RNA (solvent is hidden) is shown at its position in the unit cell.

The hairpin had 11 base pairs in the stem, two unpaired bases in the hairpin loop and two unpaired bases at the termini (Fig. 3 ▸ *b*). The stem was not longer than one helical turn, so it lacked TPS caused by helical turns. The stem of the hairpin was aligned parallel to the diagonal of the **a** × **b** face of the unit cell (Fig. 4 ▸ *a*), so the normal vectors of many of the base-pair planes were parallel to the diagonals on the **a** × **b** face of the tetragonal unit cell. These interatomic vector lead to accumulation of Patterson density along these diagonals (Fig. 4 ▸). No peaks of >5σ were found beyond the peaks owing to the adjacent base pairs, so the Patterson of the hairpin gave no evidence of TPS from helical repeats.

### 3.2. Initial *ab initio* structure determination of the dsRNA   

To determine the structure of the dsRNA, we used an almost complete diffraction data set. This data set had a resolution limit of 1.05 Å and a Wilson *B* factor of 10.6 Å^2^ (Table 1 ▸; Fig. 1 ▸ *a*). We used *SIR*2014 v.7 for structure determination. We used the modern direct-methods (MDM) protocol in *SIR*2014, which starts with the atomic composition of the RNA and a set of random phases. *SIR*2014 converted the observed structure factors (*F*s) into normalized structure factors (*E*s). The 3188 largest *E*s (|*E*_min_| = 1.716) were used to develop the phase relationships. *SIR*2014 used 300 000 structure invariants with an extended tangent formula to refine the phases of the strongest *E*s (Burla *et al.*, 2013 ▸). The phases were then extended and refined in real space by density modification. *SIR*2014 used the default parameter values in the direct-space refinement (DSR) module.

*SIR*2014 used the global phasing statistic called the ‘final figure of merit 2’ (fFOM2) to assess the promise of a phase set:
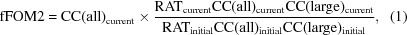


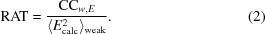
CC is the correlation coefficient between the observed and calculated normalized structure factors. The word ‘all’ means all of the normalized structure factors *E*; ‘large’ means the subset of reflections with the 70% largest *E*s and ‘weak’ means the subset of reflections with the 30% smallest *E*s. CC_*w*,*E*_ is the correlation coefficient between the largest 70% of observed *E*s and the corresponding statistical weights. The *E*_calc_ values are from the inverse Fourier transform of the current electron-density map. RAT (2) is a global figure of merit used in past versions of *SIR* (Burla *et al.*, 2002 ▸). 〈*E* ^2^_calc_〉_weak_ is the mean of the 30% of the *E*s with the weakest amplitudes.

When the fFOM2 for a trial was greater than 3.0, the trial was likely to be a success. *SIR*2014 wrote the phases and coordinates to files and then stopped. *SIR*2014 never used the remaining sets of random phases from the initial collection of 600. If the fFOM2 remained below 3.0, *SIR*2014 abandoned the phase set. *SIR*2014 selected the next phase and repeated the structure-determination protocol. The default limit of 600 phase sets was used in each phasing experiment. If all 600 phase sets failed, *SIR*2014 stopped and we tallied the phasing experiment as a failure. The correct phase set or its opposite hand was not distinguished by the fFOM2. Both hands were counted as successes because changing the hand of the phases is trivial.

The 194th random phase set for the dsRNA (Fig. 1 ▸ *b*) gave the first value (5.115) for the fFOM2 that was greater than 3.0 (Fig. 2 ▸ *b*). *SIR*2014 stopped after writing out the final coordinates and phases and left the remaining 406 phase sets untested. This 194th phase set had a low weighted mean phase error (wMPE = 23.3°; Fig. 1 ▸ *c*) when compared with the final refined structure. The final trial was reached after 9 h 56 min on a single CPU at OSCER. One processor on a late-2013 MacBook Pro laptop computer with 16 GB of RAM took a similar amount of time to reach a correct structure.

The complete RNA strand and many solvent molecules appeared in the figure of merit (FOM)-weighted *F*_o_ map that was obtained with the *ab initio* phases from *SIR*2014 (Fig. 1 ▸ *d*). *SIR*2014 placed atoms by peak picking and assigned atom types by peak height. The 31 P atoms of the one strand in the asymmetric unit were assigned correctly, but the assignment of the light C, N and O atoms had errors. Variation in the ADPs of C, N and O atoms caused overlap in the peak heights for these atoms. For example, errors are obvious in the model of a G–U wobble base pair (Fig. 1 ▸ *d*). Manual correction of this model was error-prone, so we replaced the *SIR*2014 model with a model built by *Nautilus*. We manually corrected the model from *Nautilus* with the molecular-graphics program *Coot* and refined the rebuilt model with *PHENIX* using riding H atoms and anisotropic ADPs. The final structure had a *MolProbity* clash score of less than 1 (Chen *et al.*, 2010 ▸). The final coordinates were deposited in the Protein Data Bank (Berman *et al.*, 2000 ▸).

### 3.3. Initial *ab initio* structure determination of the hairpin RNA   

**Figure 5:**
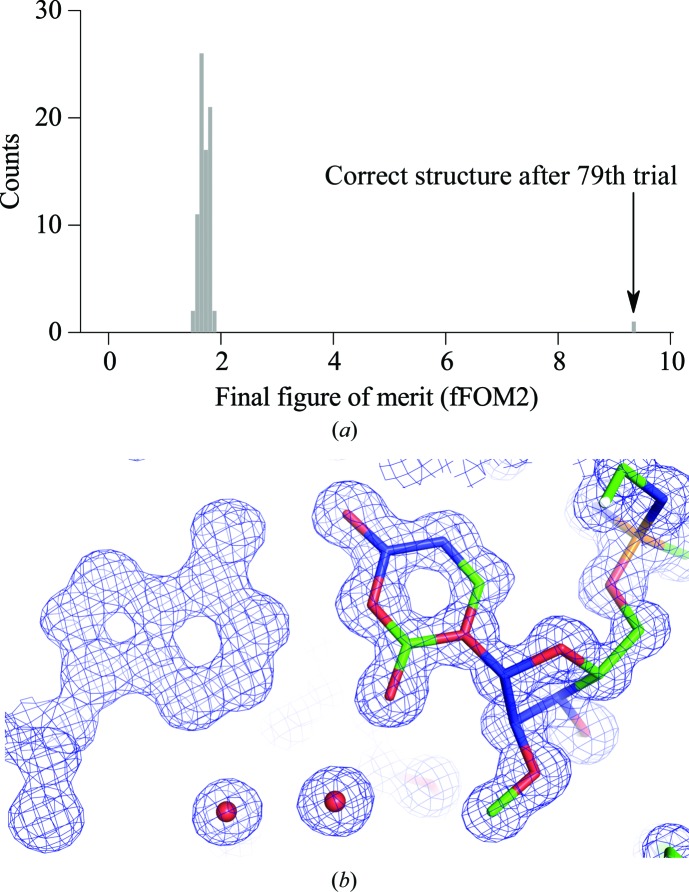
Initial structure determination of the known 27-nucleotide hairpin from random phases by direct methods using *SIR*2014. (*a*) Distribution of the fFOM2. (*b*) *F*_o_exp(*SIR*2014 phases) map.

The above procedure was also used to determine the structure of the hairpin RNA. A correct structure was reached on the 79th trial in the initial phasing experiment (Fig. 5 ▸ *a*). The fFOM2 for the correct structure was close to 10 and was almost twice the fFOM2 for the dsRNA (Fig. 1 ▸ *b*). The mean of the fFOM2s for the failed trials was closer to 2.0 compared with the mean for the dsRNA (Fig. 1 ▸ *b*). These differences in the distributions of the fFOM2s could have many causes including the absence of TPS, a slightly smaller asymmetric unit and somewhat higher resolution data for the hairpin. The *ab initio*-phased electron-density map showed the methyl group on the O2′-methyluridine at site 2650 of the hairpin RNA (Fig. 5 ▸ *b*). The light-atom assignment was also inaccurate in this structure.

### 3.4. Distributions of the number of trials before success   

**Figure 6:**
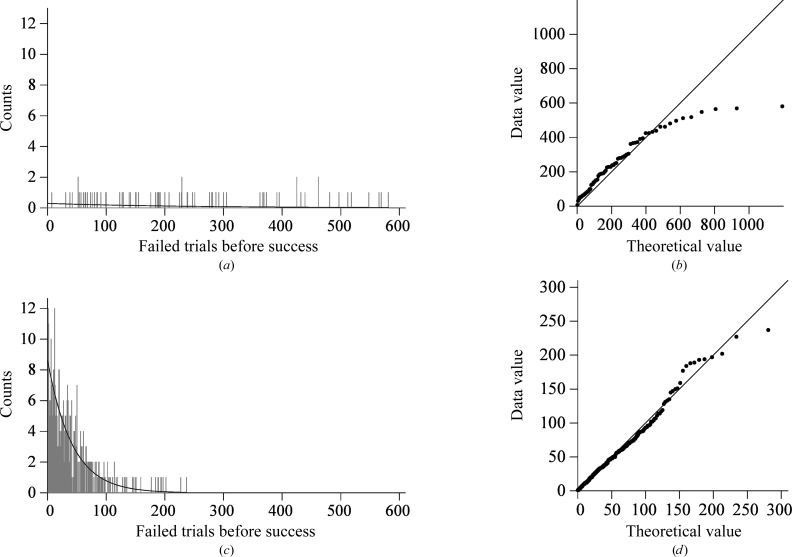
(*a*, *c*) The distribution of the number of failed trials tested (*i.e.* the number of random phase sets tested) to reach a correct structure in (*a*) 64 independent phasing experiments with the dsRNA data and (*c*) 364 phasing experiments with the hairpin data. The curves are the fitted geometric distributions. The geometric distribution has a single parameter, the probability of success in a trial. This probability the associated standard error: probability = 0.004142 ± 0.000491 for the dsRNA data and probability = 0.0321 ± 0.0012 for the hairpin data. (*b*, *d*) Geometric probability plots of the theoretical values *versus* observed data for (*b*) the dsRNA and (*d*) the hairpin RNA. The correlation coefficient was 0.95 for the dsRNA and 0.99 for the hairpin RNA. Probability computations were performed with the *MASS* package in *R* (Venables & Ripley, 2002 ▸).

We repeated the phasing experiments 91 times with the dsRNA data (Fig. 6 ▸ *a*) and 365 times with the hairpin data (Fig. 6 ▸ *c*) to characterize the distribution of the number of failed trials before success. Each phasing experiment tried up to 600 sets of random phases. 600 trial phase sets could be tested within the 48 h time limit for the batch jobs running at OSCER. We tested 17 070 different sets of random phases with the dsRNA data and 15 384 different sets of random phases with the hairpin data. Of the 91 experiments initiated for the dsRNA, 68 phasing experiments led to correct structures (Fig. 6 ▸ *a*). The arithmetic mean number of failed trials before the first successful trial was 239.4 (s.d. = 160.6). With the hairpin data, 364 of 365 phasing experiments led to correct structures (Fig. 6 ▸ *c*). The mean number of failed trials before the successful trial with the hairpin data was 41.3 (s.d. = 41.5). The geometric probability plots show that both sets of count data follow geometric distributions (Figs. 6 ▸ *b* and 6 ▸ *d*). The outliers for the dsRNA in Fig. 6 ▸(*b*) are a result of limiting the number of trials to 600. For clarity, the theoretical pmfs are shown as continuous curves instead of as impulse plots like the empirical pmfs (Figs. 6 ▸ *a* and 6 ▸ *c*). The geometric distribution has a single parameter, the probability: 0.00414 ± 0.000491 for the dsRNA and 0.0231 ± 0.0012 for the hairpin. The two empirical pmfs were also compared with the nonparametric *K*-sample Anderson–Darling test (Scholz & Stephens, 1987 ▸; Scholz & Zhu, 2015 ▸), which makes no assumptions about the distribution for the random variable. The null hypothesis that all samples come from the same population was easily rejected (*p* = 2.3016 × 10^−43^).

### 3.5. Comparison of intensity distributions   

**Figure 7:**
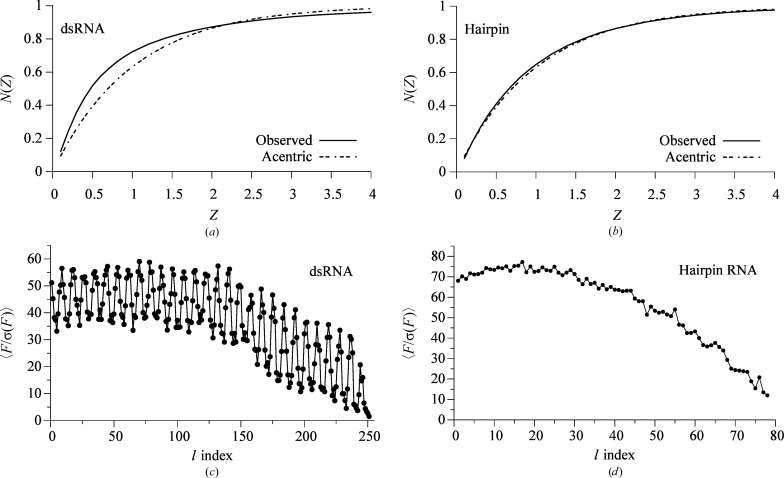
Intensity and structure-factor statistics. The cumulative distribution function (cdf) of the normalized intensities (*Z* = *E* ^2^) for the observed data (solid lines) and the Wilson acentric distribution (dashed lines) for (*a*) the dsRNA data and (*b*) the hairpin data. (*c*, *d*) Each structure factor was divided by its corrected sigma and then averaged by its *l* Miller index. (*c*) The diffraction data for the dsRNA; (*d*) the diffraction data for the hairpin RNA.

The TPS of the dsRNA was expected to distort the distribution of the intensities. We compared the empirical cumulative distribution functions (cdfs) of the normalized structure factors squared (*i.e.* |*E* ^2^| = *Z*, acentric reflections only) from the observed data with the theoretical cdf for the Wilson distribution of acentric reflections. The acentric data from the hairpin followed the theoretical acentric distribution (Fig. 7 ▸ *b*), but the cdf for the dsRNA data did not (Fig. 7 ▸ *a*). The cdf for the dsRNA data also did not follow the theoretical distribution for perfect TPS with three repetitions (data not shown; Srinivasan & Parathasarathy, 1976 ▸). This discrepancy may be caused by the imperfect TPS of the dsRNA. This imperfect TPS is reflected in the bimodal pattern of the mean structure factors when averaged by their *l* index (Fig. 7 ▸ *c*). The TPS enhanced the intensity of reflections with *l* indices of 9*n* or close to 9*n* and depressed the intensity of reflections with values of *l* = 9*n* ± 4 or 5 (Fig. 7 ▸ *c*). The hairpin data lacked the alternating pattern in the amplitudes when averaged by their *l* indices (Fig. 7 ▸ *d*).

### 3.6. Distribution of the *RELAX* vectors   

**Figure 8:**
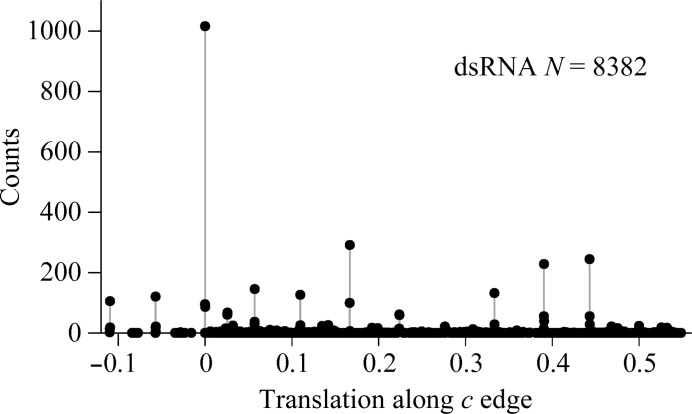
Distribution of the *z* components of the *SIR*2014 *RELAX* shift vectors for moving misplaced trial structures to the correct origin in phasing experiments with the dsRNA data in the presence of TPS.

A correct structure often developed in the wrong position in the unit cell. The *RELAX* procedure in the *SIR*2014 procedure attempted to shift the structure to the proper position. The counts of the *x* and *y* components of the shift vectors were evenly distributed between the different origins in the **a** × **b** plane of both of the unit cells (data not shown). The *z* coordinate was arbitrary for the hairpin in *P*4_3_. The *z* components of the shift vectors for the dsRNA were zero (the correct value because the molecular dyad sits on a crystallo­graphic twofold) for about one eighth of the trials but had different values for the remaining trials (Fig. 8 ▸). These non­zero values reflect the difficulty in placing the dsRNA along the *c* axis in the presence of TPS.

### 3.7. Removal of the strongest reflections   

**Figure 9:**
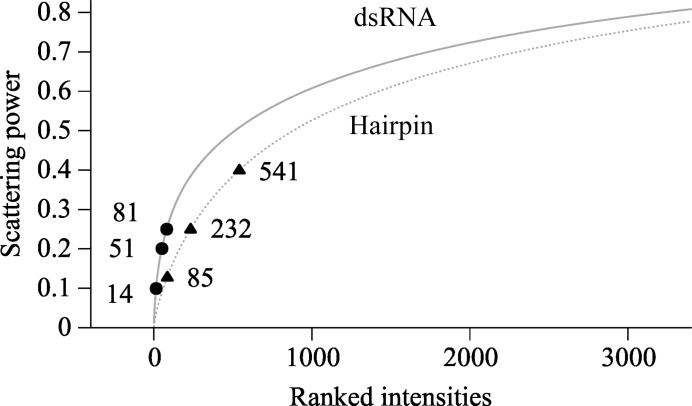
Cumulative scattering power of the diffraction data from the dsRNA (solid line) and hairpin RNA (dashed line). The quotient of the structure factor squared and the sum of the squared structure factors gave the relative contribution of a particular reflection to the total scattering power. The contribution of *F*_000_ was ignored. The points indicate the numbers of strong reflections removed in deletion data sets that tested the importance of the strongest reflections in phasing experiments.

**Figure 10:**
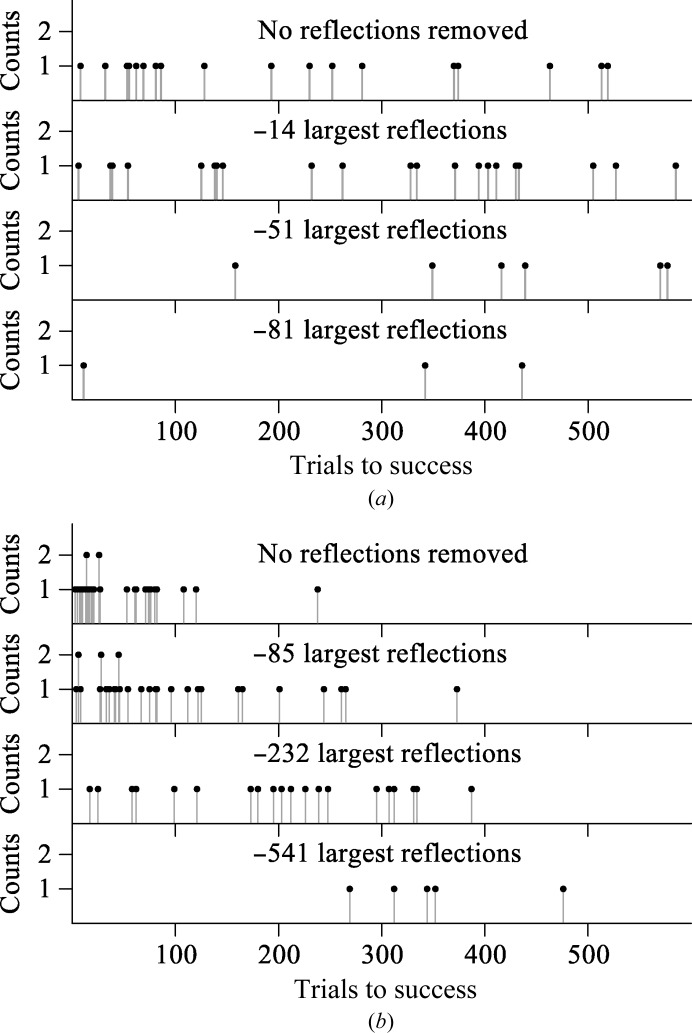
Comparison of the phasing experiments with all of the data and three deletion data sets. Each data set was used in 30 phasing experiments. (*a*) With the dsRNA (PDB entry 5da6), there were 30, 20, seven and four successes from top to bottom. (*b*) With the hairpin (PDB entry 5d99), there were 30, 30, 20 and five successes from top to bottom.

The TPS in the dsRNA data caused the strongest reflections to contribute more to the total scattering power than in the hairpin RNA data (Fig. 9 ▸). Structure determinations with the dsRNA data were expected to be more sensitive to the loss of the strongest reflections. Removal of the top 81 reflections from the dsRNA had the same effect as removing the top 232 reflections from the hairpin data (Fig. 10 ▸). The strongest reflections were more important in the dsRNA data.

## 4. Discussion   

### 4.1. Structure determination from random phases in the presence of imperfect TPS   

We report a case of *ab initio* structure determination of a dsRNA with TPS caused by the helical repeats. This intramolecular TPS was strong enough to give a bimodal structure-factor distribution but was too weak to be automatically detected by *SIR*2014. We compare this case with that of a RNA hairpin of similar size and with data of similar resolution but without TPS. Success with the dsRNA (675 non-H atoms) was achieved in 10 h using one CPU. This result was obtained with 1.05 Å resolution diffraction data from a crystal with no atoms heavier than phosphorus. We found no published evidence of a *ab initio* direct-methods structure determination of a larger nucleic acid in the absence of calcium or heavier atoms. The dsRNA is 41% larger than the previous record (Table 3 ▸).

### 4.2. Intramolecular TPS in RNA with three helical turns   

TPS usually relates copies of biopolymers within the asymmetric unit (Zwart *et al.*, 2008 ▸), but here the TPS relates helical repeats in one strand of the dsRNA. This intramolecular TPS was imperfect owing to ‘displacive’ deviations in atom positions from ideal pseudosymmetry and ‘replacive’ deviations in atomic composition owing to differences in sequence and termini structure (MacKay, 1953 ▸; Cascarano *et al.*, 1988*a* ▸,*b* ▸). The partial TPS caused high valleys in the bimodal structure factors averaged by their *l* Miller indices. More sensitive methods are needed for the automated detection of partial TPS.

### 4.3. TPS enhanced the role of the strongest reflections in phasing   

The largest *E* values give the most reliable phase relationships. The loss of only the top 1% reduced the number of successful phasing experiments with both data sets, and the dsRNA data were more sensitive to the loss of the strongest reflections than the hairpin data. The dynamic range of the diffraction intensities can be 10^7^ from crystals of dsRNA, so detector saturation is a serious issue. Guidelines for collecting complete diffraction data at atomic resolution can be found in Dauter (1999 ▸).

### 4.4. The number of trials to a correct structure   

Structure determination by *ab initio* direct methods is a stochastic process, so we repeated the phasing experiments large numbers of times (*n* > 90) for both data sets to obtain the empirical probability mass functions (pmfs) of the number of failed trials before success. The mean number of trials for the dsRNA was nearly six times larger than that for the hairpin. Both pmfs have geometric distributions, in agreement with the probability theory for Bernoulli trials, but the pmfs were statistically different in spite of similar completeness, resolution limit and size. Phasing experiments with other 32-base-pair RNAs are needed to to determine whether the pmf for the diffraction data from the dsRNA is representative of the pmfs for other 32-base-pair RNAs in the same space group. This requirement also applies to hairpin RNAs. Nonetheless, our pmfs provide benchmarks for hard and easy structure determinations of nucleic acids by *ab initio* direct methods.

### 4.5. Other *ab initio* structure-determination protocols   

Other direct-methods programs [*e.g. SHELXD* (Sheldrick, 2008 ▸) and *SnB* (Miller *et al.*, 2007 ▸)] use different phasing protocols. One or more of these programs may also succeed with the dsRNA data. The charge-flipping program *SUPERFLIP* succeeded with a case of a planar molecule of 45 atoms with intermolecular TPS (Oszlányi *et al.*, 2006 ▸), but we had no success with the dsRNA data. The programs *ARP*/*wARP* and *ACORN* can start phasing in real space with randomly placed atoms (Tame, 2000 ▸; Dodson & Woolfson, 2009 ▸), but *ACORN* failed with the dsRNA data when starting from a random atom. Success where failure occurred may still be possible by optimization of the parameters of a protocol.

## Supplementary Material

PDB reference: RNA (32-mer), 5da6

PDB reference: RNA hairpin, 5d99

## Figures and Tables

**Table 1:** Data collection and processing for dsRNA (PDB entry 5da6) Values in parentheses are for the outer shell.

Diffraction source	Beamline 7-1, SSRL
Wavelength (Å)	0.97946
Temperature (K)	100
Detector	ADSC Quantum 315r
Space group	*R*32:*H*
*a*, *b*, *c* (Å)	42.89, 42.89, 266.94
Mosaicity (°)	0.22
Resolution range (Å)	36.79–1.05 (1.10–1.05)
Total No. of reflections	355361 (41841)
No. of unique reflections	41841 (6468)
Completeness (%)	99.9 (99.9)
Multiplicity	7.9 (6.8)
〈*I*/σ(*I*)〉	20.1 (2.27)
*R*_r.i.m._	0.057 (0.440)
Overall *B* factor from Wilson plot (Å^2^)	11.3

**Table 2:** Structure refinement for structures determined *ab initio* with *SIR*2014 Values in parentheses are for the outer shell.

	U-helix dsRNA (PDB entry 5da6)	Hairpin RNA (PDB entry 5d99)
Resolution range (Å)	36.8–1.05 (1.10–1.05)	21.0–0.97 (0.98–0.97)
Completeness (%)	99.6 (99.1)	99.9 (98.6)
σ Cutoff	2.0	2.0
No. of reflections, working set	42604	38814
No. of reflections, test set	2337	2029
Final *R*_cryst_	0.1301	0.105
Final *R*_free_	0.1568	0.120
Cruickshank DPI†	0.025	0.018
No. of non-H atoms		
RNA	675	580
Ligand	1	14
Water	236	183
R.m.s. deviations		
Bonds (Å)	0.005	0.006
Angles (°)	1.0	1.2
Average *B* factor for RNA (Å^2^)	13.98	10.78
*MolProbity* clash score	0.0	0.0

† Computed using *Online_DPI* (Kumar *et al.*, 2015 ▸).

**Table 3:** Previously unknown nucleic acid crystal structures determined by direct methods starting from random phases

Molecule	Non-H†	*d*_min_ (Å)	*Z* > 19	Program	PDB code	Reference
10 nt DNA	408	0.83	0	*SnB*	1dpl	Egli *et al.* (1998 ▸)
11 nt DNA/RNA	414	1.15	3 Ca^2+^	*SnB*	1g4q	Han (2001 ▸)
11 nt RNA	478	1.0	0	*SHELXD*	4jrd	Safaee *et al.* (2013 ▸)
12 nt DNA	243	0.75	0	*SHELXD*	4ocb	Luo *et al.* (2014 ▸)
32 nt RNA	675	1.05	0	*SIR*2014	5da6	This work

† The number of non-H nucleic acid atoms in the asymmetric unit.
